# Supplementary material for: Accelerating the prediction of CO2 capture at low partial pressures in metal-organic frameworks using new machine learning descriptors
Source: Commun Chem. 2023 Oct 3;6:214. doi: 10.1038/s42004-023-01009-x (PMC10547688; doi:10.1038/s42004-023-01009-x)
Supplement: Supplementary file 2 — Supplementary Information [file 42004_2023_1009_MOESM2_ESM.pdf]

Supplementary Information for:
Accelerating the Prediction of CO_2 Capture at Low Partial Pressures in Metal–Organic Frameworks using New Machine Learning Descriptors

Ibrahim B. Orhan, Tu C. Le, Ravichandar Babarao and Aaron W. Thornton

Supplementary Discussion

The EPoCh Descriptor

The derivation of the EPoCh descriptor consisted of simulating point charges (massless atoms where van der Waals were excluded) at varying pressures to identify the effects of charge on CO_2 uptake. The surface was fitted using a least-squares fit utilising high enough powers of the pressure and charge parameters that there was good agreement between the results obtained through the polynomial and the results of the simulations. More simulations were run at the lowest pressures to obtain better characterisation of uptake at those settings. Since this polynomial was fit to the pressure- and charge-ranges indicated in Figure S1.1 below, for pressures above 10,000 Pa, we recommend choosing either 10,000 Pa directly or a lower pressure to evaluate partial charges within a framework such as 40 Pa where the resulting descriptors are shown to have considerable importance in ML models.

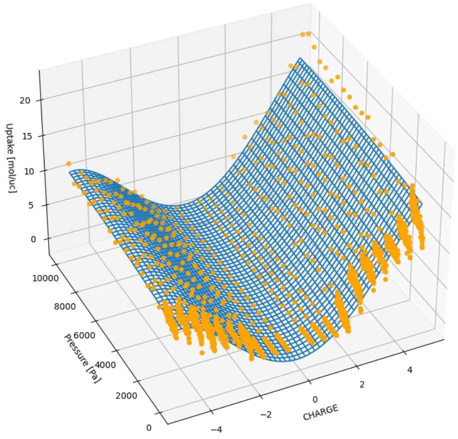

Supplementary Figure 1: Point charge simulation results. *Point charge simulation results (orange) and the surface plotted by the equation fitted to the results*

$$f(Q,p) = \alpha_1 Q + \alpha_2 Q^2 + \alpha_3 Q^3 + \alpha_4 Q^4 + \alpha_5 Q^5 + \alpha_6 Q^6 + \alpha_7 Q^7 + \alpha_8 p + \alpha_9 p^2 + \alpha_{10} p^3 + \alpha_{11}$$

Where:

a_1= -4.0148e-01
a_2= 9.5868e-01
a_3= 2.0246e-01
a_4= -3.2578e-02
a_5= -1.4451e-02
a_6= 5.0194e-04
a_7= 3.1280e-04
a_8= 1.1229e-03
a_9= -2.0035e-07
a_{10}= 1.1667e-11
a_{11}=-6.2865e-01

(Supplementary equation 1)

Since the results are in terms of molecules of CO_2 within the unit cell, to convert to mol cm^{-3} a conversion factor, *k*, can be used. k = 0.0002075, obtained by multiplying with the inverse of Avogadro's number with 10^{24} (converting A^3 to cm^3) and dividing by 8000 (unit cell volume in A^3).

EPoCh Descriptors (https://github.com/ibarisorhan/EPoCh-Descriptors), and Atom Type and Chemical Descriptors (https://github.com/ibarisorhan/MOF-Features) are available online.

Time weighted metrics

Reducing the computational time to evaluate each MOF is one of the major contributing factors to using ML over direct simulation. While it is clear that the Henry coefficient is effective in the creation of accurate ML models when predicting CO_2 capture, it has the drawback of requiring considerable time to calculate. The feasibility of using the Henry coefficient diminishes as datasets grow to the hundreds of thousands or even millions of candidate materials.

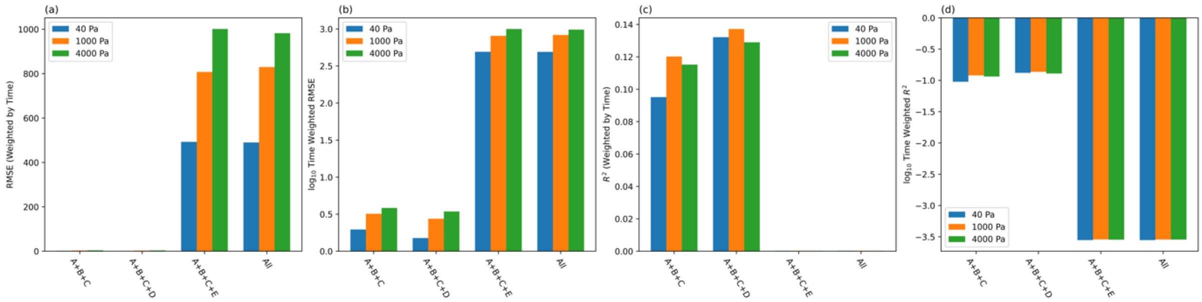

Supplementary Figure 2: Time weighted metrics. Time weighted metrics for (a)(b) RMSE and (c)(d) R2, where RMSE was weighted by multiplying with the mean time necessary for gathering each descriptor and R2 was weighted by multiplying with the mean time necessary for gathering each descriptor, based on timings of the Anion Pillared MOFs dataset.

When incorporating time into the metrics, we can see that the performance of using the Henry coefficient is reduced due to the slowed evaluation times when a complete dataset is gathered. In contrast, the rapidity of models without this descriptor are highlighted in the time-weighted metrics.

Evaluating the Performance and Robustness of the Machine Learning Model

The robustness of the machine learning model was evaluated by looking at the performance of predictions made at pressures which the model had not previously seen. In addition to the descriptors used to train each of the pressure-specific models, "pressure" was added as an additional descriptor.

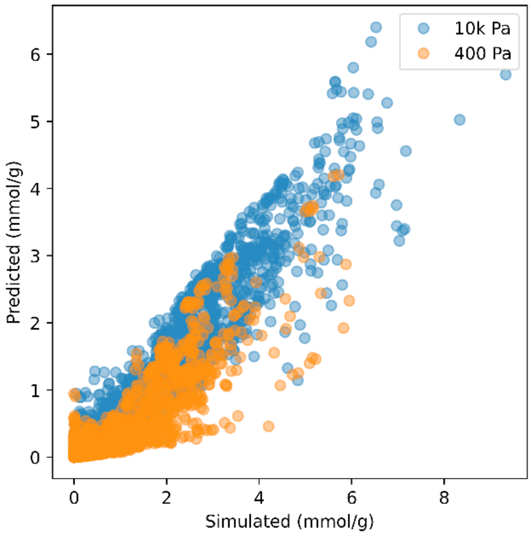

Supplementary Figure 3: Predicted uptakes vs simulated uptakes for pressures unseen to the ML model.

Supplementary Figure 3 demonstrates the agreement between the uptakes determined through GCMC and those predicted through the ML model. At 400 Pa the model yielded an R^2 of 0.54 while at 10,000 Pa the R^2 was 0.72 when the Henry coefficient was excluded from the dataset.

Supplementary Table 1. Performance metrics of the model at 40 Pa, 1,000 Pa, and 4,000 Pa

Pressure (Pa)	Features	R2 (Train)	RMSE (Train)	R2 (Test)	RMSE (Test)
40	A+B+C	0.9331	0.1363	0.5143	0.3629
40	A+B+C+D	0.9532	0.114	0.7147	0.2781
40	A+B+C+E	0.9913	0.0491	0.9164	0.1505
40	All	0.9912	0.0494	0.9172	0.1498
1000	A+B+C	0.9446	0.221	0.6499	0.592
1000	A+B+C+D	0.9624	0.182	0.7424	0.5079
1000	A+B+C+E	0.991	0.089	0.9391	0.2469
1000	All	0.9909	0.0894	0.9358	0.2536
4000	A+B+C	0.9485	0.2821	0.6232	0.7096
4000	A+B+C+D	0.9649	0.233	0.6976	0.6357
4000	A+B+C+E	0.9902	0.1233	0.9299	0.306
4000	All	0.9903	0.1227	0.9325	0.3003

Supplementary Note 1: CO_2 Molecule Parameters

S1.1. Molecule Definition File: CO_2

```
# critical constants: Temperature [T], Pressure [Pa], and Acentric factor [-]
304.1282
7377300.0
0.22394
#Number Of Atoms
 3
# Number of groups
1
# CO2-group
rigid
# number of atoms
3
# atomic positions
0 O_co2    0.0        0.0        1.149
1 C_co2    0.0        0.0        0.0
2 O_co2    0.0        0.0        -1.149
# Chiral centers Bond  BondDipoles Bend  UrayBradley InvBend  Torsion Imp. Torsion Bond/Bond Stretch/Bend
Bend/Bend Stretch/Torsion Bend/Torsion IntraVDW IntraCoulomb
         0  2       0  0      0    0    0      0     0      0     0       0      0    0      0
# Bond stretch: atom n1-n2, type, parameters
0 1 RIGID_BOND
1 2 RIGID_BOND
# Number of config moves
0
```

S1.2. Pseudo Atoms: CO_2

```
#type      print   as    chem  oxidation   mass        charge   polarization B-factor
radii  connectivity anisotropic anisotropic-type   tinker-type
C_co2      yes     C     C     0           12.0        0.6512   0.0          1.0
0.720  0            0           relative           0
O_co2      yes     O     O     0           15.9994    -0.3256   0.0          1.0
0.68   0            0           relative           0
~
```

S1.3. Van Der Waals Parameters: CO_2

```
#general rule for shifted vs truncated
shifted
# general rule tailcorrections
no
# number of defined interactions
2
# type interaction, parameters.
C_co2 lennard_jones   29.933       2.745
O_co2 lennard-jones   85.671       3.017
# general mixing rule for Lennard-Jones
Lorentz-Berthelot
```

Supplementary Note 2: H_2O (TIP4P) Parameters

S2.1. Molecule Definition File: H_2O

```
# critical constants: Temperature [T], Pressure [Pa], and Acentric factor [-]
647.14
22055000.0
0.3449
#Number Of Atoms
4
# Number of groups
1
# main-group
rigid
# number of atoms
4
# atomic positions
0 O_w              0.0000   0.0000   0.0000
1 H_w             -0.7570   0.5859   0.0000
2 H_w              0.7570   0.5859   0.0000
3 M_w              0.0000   0.1546   0.0000
# Chiral centers Bond  BondDipoles Bend  UrayBradley InvBend  Torsion Imp. Torsion
Bond/Bond Stretch/Bend Bend/Bend Stretch/Torsion Bend/Torsion IntraVDW IntraCoulomb
               0    3            0    0            0       0        0            0
0            0         0               0            0        0            0
# Bond stretch: atom n1-n2, type, parameters
0 1 RIGID_BOND
0 2 RIGID_BOND
0 3 RIGID_BOND
# Number of config moves
0
```

S2.2. Pseudo Atoms: H_2O

```
#type      print   as    chem  oxidation   mass        charge   polarization B-factor
radii  connectivity anisotropic anisotropic-type   tinker-type
H_w         yes     H     H     0           1.008       0.5564   0.0          1.0
0.7    0            0           relative           0
O_w         yes     O     O     0           15.9994     0.0000   0.0          1.0
0.68   0            0           relative           0
M_w         no      M     -     0           0.0        -1.1128   0.0          1.0
0.7    0            0           relative           0~
```

S2.3. Van Der Waals Parameters: H_2O

```
#general rule for shifted vs truncated
shifted
# general rule tailcorrections
no
# number of defined interactions
3
# type interaction, parameters.
H_w   none
O_w   lennard-jones    93.2   3.1589
M_w   none
# general mixing rule for Lennard-Jones
Lorentz-Berthelot
```

Supplementary Tables: Effects of Humidity

Supplementary Table 2.1. Hydrophobic MOFs with an uptake greater than 1 mmol g^{-1} at 40Pa

MOF	Henry Coeff CO_2	Henry Coeff H_2O	Uptake (mmol g^{-1})	PLD (Å)	LCD(Å)	LFPD(Å)
SIFSIX_3_Cu	0.745689	0.605362	2.4863981803	3.07218	4.34233	4.34233
BUSQIQ	0.123034	0.0592101	2.2419801206	3.72721	4.44057	4.4272
SIFSIX_3_Ni	0.125899	0.0294069	1.8237632712	3.16641	4.46528	4.46528
GeFSIX_3_Ni	0.104961	0.050871	1.57366658	3.18971	4.45386	4.45386
SIFSIX_3_Zn	0.0743006	0.0441128	1.4640006039	3.27027	4.59141	4.59141
SIFSIX_3_Co	0.0623964	0.0159088	1.3283975054	3.22223	4.54234	4.54234
GeFSIX_3_Co	0.0426567	0.0271157	1.0335770258	3.27308	4.55994	4.55994

Supplementary Table 2.2. Hydrophobic MOFs with an uptake greater than 1 mmol g^{-1} at 1000Pa

MOF	Henry Coeff CO2	Henry Coeff H2O	Uptake (mmol g^{-1})	PLD (Å)	LCD(Å)	LFPD(Å)
LOGBEO	0.0113216	0.000882823	4.8410786778	3.5444	8.32475	8.32475
LOFZUB	0.00907767	0.000642098	4.5853732155	3.56551	8.36948	8.36948
HITXUE	0.00558193	0.000570237	4.2231019822	3.4579	7.01987	7.01987
BUSQIQ	0.123034	0.0592101	4.2055357251	3.72721	4.44057	4.4272
SIHLUQ	0.00852057	0.000152534	4.0141040747	3.06642	6.79694	6.79694
MOPKEJ	0.00628158	0.000165559	3.0163199622	3.52649	5.74838	5.74349
SIFSIX_3_Cu	0.745689	0.605362	2.706803676	3.07218	4.34233	4.34233
SIFSIX_3_Ni	0.125899	0.0294069	2.7023505646	3.16641	4.46528	4.46528
SIFSIX_3_Co	0.0623964	0.0159088	2.6455684945	3.22223	4.54234	4.54234
SIFSIX_3_Zn	0.0743006	0.0441128	2.6191466152	3.27027	4.59141	4.59141
SIFSIX_3_Fe	0.0312209	0.0110433	2.5629801303	3.32602	4.65996	4.65995
GeFSIX_3_Ni	0.104961	0.050871	2.3926765156	3.18971	4.45386	4.45386
VOFFIVE_3_Ni	0.0240768	0.0227419	2.3538740848	3.20063	4.47081	4.47081
GeFSIX_3_Co	0.0426567	0.0271157	2.320274928	3.27308	4.55994	4.55994
GeFSIX_3_Fe	0.0242447	0.0217469	2.2478957535	3.36106	4.65526	4.65526
VOFFIVE_3_Co	0.0127455	0.012228	2.1637762116	3.29176	4.57515	4.57515
GUMDEZ	0.00367764	0.000333701	2.1038250588	4.82333	5.2713	5.25709
LOKPEG	0.00284411	0.000132663	2.0715243348	2.49075	4.37004	4.09581
VOFFIVE_3_Fe	0.00742917	0.00574364	1.9826267503	3.29395	4.56637	4.56637
MILQIJ	0.00257205	0.00137977	1.8254192164	4.0045	4.64027	4.64027
PARMIG	0.00388615	4.45183e-05	1.7849171881	4.27949	4.62483	4.61822
QUZZES	0.00222282	0.000366743	1.6843540245	3.4268	5.06119	5.05316
ADIQEL	0.0018242	7.87791e-05	1.5091150277	3.91364	4.43355	4.39291
BFFIVE_2_Ni_i	0.00126223	0.00104591	1.3174861356	3.81595	4.56787	4.55937
XEQLEM01	0.00381328	8.6476e-05	1.2888576153	2.46232	4.50974	4.44269
EZEQEH	0.00175545	0.00151357	1.2570636907	4.37059	4.87284	4.87284
XEQLEM	0.00357333	9.84775e-05	1.2538930479	2.48011	4.53865	4.51762
QIWDOR	0.00293968	0.00107896	1.2242326546	4.0548	4.57135	4.57135
AGESIP	0.00192851	0.000302274	1.1796824306	3.05111	3.66544	3.64809
HIHFOV	0.00151662	0.000225504	1.0629787958	3.46095	4.86314	4.86114
TATSIS	0.00261778	9.20079e-05	1.0356189495	2.43302	4.50814	4.40586
VIQQUJ	0.00118222	0.000484268	1.0195516672	3.89522	4.62845	4.6169

Supplementary Table 2.3. Hydrophobic MOFs with an uptake greater than 1 mmol g^{-1} at 4000Pa

MOF	Henry Coeff CO_2	Henry Coeff H_2O	Uptake (mmol g^{-1})	PLD (Å)	LCD(Å)	LFPD(Å)
SIHLUQ	0.00852057	0.000152534	5.3327880484	3.06642	6.79694	6.79694
BUSQIQ	0.123034	0.0592101	4.6281051712	3.72721	4.44057	4.4272
MOPKEJ	0.00628158	0.000165559	4.3404638235	3.52649	5.74838	5.74349
BFFIVE_2_Ni_i	0.00126223	0.00104591	4.1967777788	3.81595	4.56787	4.55937
TOXMUQ	0.000760845	0.000106837	3.9083199561	6.74832	6.89787	6.89787
QUZZES	0.00222282	0.000366743	3.885363593	3.4268	5.06119	5.05316
XUPSAE	0.000618628	0.000102679	3.5365699393	6.86151	6.99773	6.99773
MILQIJ	0.00257205	0.00137977	3.3448863084	4.0045	4.64027	4.64027
ADIQEL	0.0018242	7.87791e-05	3.3410449687	3.91364	4.43355	4.39291
TOXNEB	0.000561185	7.82482e-05	3.2347183248	6.86576	7.00092	7.00092
GUMDEZ	0.00367764	0.000333701	3.2272798887	4.82333	5.2713	5.25709
TOXNIF	0.000561666	8.79593e-05	3.2109301917	6.82273	6.95996	6.95996
BFFIVE_2_Fe_i	0.000917808	4.24711e-05	2.8835114937	3.9402	4.82079	4.24216
PARMIG	0.00388615	4.45183e-05	2.8709935508	4.27949	4.62483	4.61822
LOKPEG	0.00284411	0.000132663	2.7766238613	2.49075	4.37004	4.09581
SIFSIX_3_Ni	0.125899	0.0294069	2.7459162913	3.16641	4.46528	4.46528
SIFSIX_3_Co	0.0623964	0.0159088	2.7299896817	3.22223	4.54234	4.54234
SIFSIX_3_Fe	0.0312209	0.0110433	2.7271462237	3.32602	4.65996	4.65995
SIFSIX_3_Cu	0.745689	0.605362	2.7196948051	3.07218	4.34233	4.34233
SIFSIX_3_Zn	0.0743006	0.0441128	2.6893310853	3.27027	4.59141	4.59141
VIQQUJ	0.00118222	0.000484268	2.6710568388	3.89522	4.62845	4.6169
VOFFIVE_3_Ni	0.0240768	0.0227419	2.5399041296	3.20063	4.47081	4.47081
EZEQEH	0.00175545	0.00151357	2.5019941646	4.37059	4.87284	4.87284
VOFFIVE_3_Co	0.0127455	0.012228	2.4800919183	3.29176	4.57515	4.57515
GeFSIX_3_Ni	0.104961	0.050871	2.4393030695	3.18971	4.45386	4.45386
VOFFIVE_3_Fe	0.00742917	0.00574364	2.4281779904	3.29395	4.56637	4.56637
GeFSIX_3_Co	0.0426567	0.0271157	2.4207211441	3.27308	4.55994	4.55994
GeFSIX_3_Fe	0.0242447	0.0217469	2.4104910022	3.36106	4.65526	4.65526
XAVFEH	0.000678632	0.000172052	2.2901470078	2.81214	3.17223	3.03202
QOZDOY	0.000897604	0.000589713	2.2545330825	4.6547	5.24337	5.23868
HIHFOV	0.00151662	0.000225504	2.1454820161	3.46095	4.86314	4.86114
YIWBEN	0.000767013	2.28564e-05	2.1405913035	3.94622	5.41707	5.41707
MIMVEJ	0.00114602	0.000147566	2.1179036213	4.2729	4.69686	4.6002
VICDOC	0.000884085	1.36911e-05	2.0867048903	4.08181	4.65057	4.52458
HOQMIL01	0.000815611	0.000803032	1.998092652	4.83187	5.56977	5.56337
BOVHEB	0.000825759	0.000545775	1.9965629253	4.22597	5.76843	5.76843
HOQMIL02	0.000780769	0.000776438	1.992666995	4.88733	5.63983	5.6271
XADGAM	0.00153532	0.000166089	1.9908832971	3.92059	5.01478	5.01478
VALZIU	0.000927867	0.000590168	1.9213731405	3.39828	4.40381	4.40103
IQINAZ	0.000947749	9.38069e-05	1.9169493699	3.30446	4.8626	4.8626
AGESIP	0.00192851	0.000302274	1.8570029842	3.05111	3.66544	3.64809
WEBREC	0.000591522	0.000174342	1.8344491168	3.77679	5.39308	5.39069
XEJTIR	0.000889532	5.20244e-05	1.7840595194	2.90436	4.61538	4.5627
MUVGOA	0.000599342	9.68353e-06	1.7697712492	3.51534	5.01082	4.91072
MEJQEZ	0.000656452	0.000205999	1.766047504	3.56716	4.67231	4.61853
FOJKIA	0.000448069	0.00035344	1.754441099	4.1527	5.14315	5.13081
QIWDOR	0.00293968	0.00107896	1.7523689639	4.0548	4.57135	4.57135
XUNSIK	0.000578369	1.29879e-05	1.7320005614	3.7899	4.24603	4.24027
UFUNIS	0.00074248	0.000141545	1.7237673048	3.76104	4.47498	4.47498
TAKTIL	0.00032313	0.000111906	1.7219870799	6.9712	7.09237	7.09237
LUFQUZ	0.000556049	0.000101838	1.7179599664	3.71299	4.20915	4.20915
LUFQUZ01	0.000543412	0.000104492	1.702997615	3.66293	4.13433	4.13433
LUDKUS	0.00138019	5.83115e-05	1.6961377146	4.22525	6.94763	6.94763
XUNRUV	0.000710688	4.86726e-05	1.6929197494	3.15718	4.49215	4.4842
XEQLEM01	0.00381328	8.6476e-05	1.6780664666	2.46232	4.50974	4.44269
MUVHAN	0.000558529	8.40222e-06	1.6766786984	3.68466	5.16934	5.16934
BFFIVE_14_Fe_i	0.00058714	0.000422683	1.6738077537	3.16575	4.56737	4.37256
HIMSAY	0.000567005	8.27898e-06	1.67246304	3.64697	5.14732	5.14732
XEQLEM	0.00357333	9.84775e-05	1.6708484962	2.48011	4.53865	4.51762
NADZID	0.000611682	0.000102574	1.6563146421	4.70268	5.01289	4.9859
FUBNOH	0.000680604	0.000520866	1.6325379978	3.53828	4.81179	4.81179
MUVGUG	0.00055429	8.72275e-06	1.6255013168	3.82713	5.28653	5.27809
POLYEW	0.000710132	4.53001e-05	1.6108990369	3.57055	5.12248	5.12248
HAWZEM	0.00059101	4.58431e-05	1.5982044097	3.71581	4.83764	4.79379
MUVHER	0.000527231	8.77506e-06	1.5913949126	3.72392	5.22081	5.21603
UXUZAP	0.00054809	0.000174819	1.5838720787	3.59621	4.80114	4.78433
OSAXAI	0.00100435	7.44678e-05	1.5833685955	3.49959	4.37814	4.35987
BENXUP	0.00052288	0.000222033	1.5680559958	3.40515	4.05291	4.05207
WALCET	0.00076699	0.000304489	1.5512024537	3.07005	4.22232	4.2126
MUDKOM	0.000780097	0.00067447	1.5488368009	2.83822	4.19059	4.17388
VIYZIO	0.00105594	0.000141473	1.5486491065	2.966	4.31178	4.31178
QERZUI01	0.000648454	0.000415362	1.5420164997	3.51691	4.39138	4.39138
VIYZIO01	0.00104867	0.000132836	1.5189593832	2.96579	4.31487	4.21111
XUNGOD	0.000457507	1.3072e-05	1.511112174	3.81361	5.27654	5.27654
WOGHEG	0.000668934	7.98267e-05	1.5066595931	3.00128	4.94866	4.86778
NEVVEQ	0.000553332	4.57508e-05	1.4871697349	3.79614	4.80581	4.80581
WALBOC02	0.0006955	0.000388656	1.4792112721	2.98726	4.18643	4.18643
PEYPIU	0.00111025	0.000221076	1.4702535161	3.52379	4.71364	4.71364
SOSSEA	0.000734494	1.42265e-05	1.4556792072	3.46037	4.66033	4.6584
MAKXAZ	0.00049483	0.000191889	1.4476350595	4.82882	5.07611	5.06464
TATSIS	0.00261778	9.20079e-05	1.4448410769	2.43302	4.50814	4.40586
XENMIO	0.00104515	1.12072e-05	1.4435190558	3.44483	4.24503	4.24503
CETVAA	0.00182709	0.000760706	1.4031405114	3.28639	4.38981	4.38981
UKUCAF	0.000539545	4.77133e-05	1.3993490844	4.4286	7.2141	6.87747
GIMSUS	0.00118321	9.22227e-05	1.3989317294	3.17204	4.94182	4.94182
XENMOU	0.000938908	1.10238e-05	1.3967643565	3.45513	4.25239	4.25142
UVARIT	0.000988913	0.000724201	1.3896197827	3.19439	4.78015	4.66439
XUNHAQ	0.000408387	9.18988e-06	1.3891998985	3.93895	4.43369	4.41861

BUKYAJ	0.000375097	4.28167e-05	1.3870132734	3.53964	5.10209	5.09477
XUNGUJ	0.000418636	9.05993e-06	1.3863382595	4.05224	4.49594	4.48031
XUNSAC	0.000432885	3.77624e-05	1.3818809912	3.78154	4.78336	4.77852
FUWXOL	0.000459039	1.02366e-05	1.3762076459	4.4326	4.75051	4.72869
EREFEN01	0.000394811	6.71268e-05	1.3710064116	3.86529	5.01639	5.00707
KINDIW	0.00081359	0.000160187	1.3679783698	3.36633	4.37637	4.37637
MEJQID	0.00042641	0.000136716	1.3657995193	3.64927	4.92572	4.86981
IJASIW	0.000791347	0.000153395	1.3637512549	3.35746	4.37434	4.37434
OTOVAW	0.000749991	0.000308133	1.3600955213	3.41338	4.51109	4.48101
ESIDOB	0.000365077	6.05655e-05	1.3564296869	4.12899	5.0954	5.08183
MATTOR	0.000536526	0.000112563	1.3483525117	3.89724	5.58509	5.58509
WALBOC	0.000600343	0.000444359	1.3471051037	2.95686	4.17238	4.17238
MEJQOJ	0.000410502	0.000207251	1.3219399984	3.67535	5.04317	4.92701
GAYGAQ	0.000434253	5.50099e-05	1.3187843202	4.6154	5.41176	5.41176
SIVVAW	0.00101012	6.9663e-05	1.3059188227	3.23479	5.08743	5.07492
TUPDOZ	0.000458682	1.64772e-05	1.2984902759	4.4419	4.88368	4.87874
RURPAW	0.000454926	5.49625e-05	1.2976292454	3.57847	4.26453	4.26352
MOTKAI	0.000387422	0.000231579	1.2975756341	2.62541	4.11967	4.11967
BOWQAG	0.000747452	0.000229274	1.2783152469	2.72062	3.45041	3.39445
OJIWIO	0.000481477	0.000155155	1.2763074778	3.98448	4.61078	4.61078
OKAZAD	0.00053208	8.10324e-06	1.2685987983	3.77862	4.45701	4.21772
BFFIVE 13 Co i	0.000420142	0.000219867	1.267761562	3.87373	4.39234	4.39234
OKAYOQ	0.000539942	7.8107e-06	1.2599902859	3.73339	4.43848	4.16809
YUNJUO	0.000501301	7.88583e-06	1.2540867644	3.63152	4.39479	4.30622
YUNKAV	0.000516133	7.00652e-06	1.2499372383	3.79299	4.51501	4.16091
OKAYUW	0.000529913	8.03229e-06	1.2470618032	3.73093	4.44294	4.20318
YUZCED	0.000652172	2.12007e-05	1.2393792889	3.01611	5.20528	5.20528
EQERIC	0.000303864	1.41741e-05	1.2348202101	5.10575	5.65308	5.50654
JODFOZ	0.000674492	0.000148281	1.2242340865	3.19319	4.69106	4.60847
DEGJIK	0.000452954	1.18394e-05	1.2198862999	3.83146	4.48185	4.48185
KAXQIL	0.0010242	1.38689e-05	1.1995868832	3.76819	5.12579	5.12409
QUXQUX	0.000988581	1.3317e-05	1.1975192984	3.8322	5.17149	5.17149
JUWBIO	0.000410202	0.00032896	1.1898404612	2.88692	4.3796	4.3796
QUXRAE	0.0010328	1.4254e-05	1.1888340306	3.68439	5.03286	5.03092
XEJXAN	0.000685493	4.42595e-05	1.1886928554	3.60567	4.27901	4.27901
QUXRUY	0.00105486	1.47143e-05	1.1872605285	3.57488	4.90652	4.90652
OKAYEG	0.00108491	1.43398e-05	1.1848526317	3.49488	4.8581	4.85158
QUWYEO	0.00105796	1.38793e-05	1.1843973698	3.64708	4.99055	4.98453
QUXREI	0.00102379	1.42986e-05	1.183061834	3.67388	5.01673	5.01152
LIDZUV	0.000972603	1.43437e-05	1.1828591593	3.77339	5.12552	5.09909
XUNSOQ	0.000336621	1.07962e-05	1.1813577323	4.41529	5.47061	5.45212
QUXROS	0.00102095	1.37843e-05	1.1812180989	3.66108	5.0019	4.99589
OTIPAK	0.000337836	0.00016407	1.178797921	5.10341	5.73499	5.72616
OKAYAC	0.000997301	1.45904e-05	1.1747254491	3.57241	4.90891	4.90891
HURGUX	0.000425228	0.000156296	1.1725626366	3.84254	4.97475	4.97057
PESTUD	0.000598894	4.27218e-05	1.1697786744	3.77014	4.29451	4.29451
OKAYIK	0.000985498	1.55045e-05	1.1622881779	3.56465	4.91102	4.89955
MEKDUC	0.000435329	9.84083e-05	1.1487283221	3.68888	4.94918	4.93711
QUXRIM	0.000956291	7.65739e-06	1.1377211767	4.31307	4.75182	4.74788
QEJYIP	0.000310476	0.0001612	1.1355501727	3.44771	5.79435	5.79373
JASNAT	0.000322955	5.49798e-05	1.125565724	3.9718	4.75713	4.75713
IGUVIR	0.000401602	2.43036e-05	1.1211036153	3.48765	5.80337	5.78397
FURFOP	0.000336991	0.000119717	1.1200072779	3.56745	4.93179	4.93179
PEPKUR	0.000522446	2.75688e-05	1.1129724133	3.85266	4.38796	4.37809
ISACIP	0.00113734	3.69611e-05	1.1121950852	3.32925	4.48211	4.47549
ZAXWEC	0.00135706	7.03543e-05	1.1090416306	2.78456	4.15244	4.07396
XAJQAC	0.000472816	4.377e-05	1.0964956493	7.41664	8.58247	8.57951
EDADIX	0.00047192	0.000178577	1.095114827	3.86408	4.60779	4.60779
IDUDIW	0.000264472	0.000116062	1.0912918811	3.47826	4.39873	4.31195
YUZCAZ	0.000493597	5.02198e-05	1.0890866276	2.90036	5.49514	5.49012
PIZRUN	0.000352916	0.000130251	1.0820826862	4.42309	5.37732	5.35351
QOKCID	0.000803957	3.18035e-05	1.0805029844	3.83184	4.28256	4.28256
YUZBUS	0.000488277	4.84661e-05	1.0787858762	2.86119	5.52385	5.51891
OTEGUR	0.000218559	4.68343e-05	1.078324879	7.1769	7.28809	7.28809
PIHNUQ	0.000651265	0.000210406	1.0762307479	3.61556	4.33002	4.33002
FOCZAA	0.000388429	1.5084e-05	1.0560018043	4.44876	4.86095	4.8459
DOYBEA	0.000270883	4.88096e-05	1.0551245072	5.37899	5.83429	5.82078
FOFCOU	0.000381124	1.56355e-05	1.0546213184	4.4269	4.8597	4.8597
PIZQUM	0.000341775	9.93587e-05	1.053916348	4.39999	5.35494	5.35201
YICREI	0.000255449	0.000182034	1.0485731694	5.57464	5.84848	5.83375
YUZBIG	0.000459612	4.53002e-05	1.0462527458	2.87444	5.54778	5.54729
IGUTUB	0.000359593	1.91034e-05	1.0414784025	3.38592	5.90315	5.90315
ZERQOE	0.000916735	7.6096e-06	1.0383147652	4.02903	4.4965	4.49472
GIRHEW	0.000389558	2.74759e-05	1.0364000295	3.3107	4.31665	4.31665
CUVGOQ	0.000250263	5.05528e-05	1.0155798456	4.5385	5.59653	5.59247
CUVGIK	0.000235272	0.000226097	1.0139767614	5.06086	6.25146	6.0647
QAVWAN	0.000287079	5.24661e-05	1.0111363152	4.72318	6.35648	6.35648
SIVWUR	0.000933635	9.5969e-06	1.006111587	4.03318	4.87776	4.87776
GEWXAJ	0.000591207	0.000402416	1.0030410244	4.49912	4.80454	4.79369
