# Supplementary material for: Accelerating the prediction of CO2 capture at low partial pressures in metal-organic frameworks using new machine learning descriptors
Source: Commun Chem. 2023 Oct 3;6:214. doi: 10.1038/s42004-023-01009-x (PMC10547688; doi:10.1038/s42004-023-01009-x)
Supplement: Supplementary file 3 — Description of Additional Supplementary Files [file 42004_2023_1009_MOESM3_ESM.pdf]

Description of Additional Supplementary Files

File name: Supplementary Data 1

Description: 12637 MOFs with predicted uptake and descriptors
